# News and Gossip

**Published:** 1870-09

**Authors:** 


### News and Gossip.

Yale College records show that twenty-five per cent. of its students never marry. Prof. Loomis, of Harvard University, (*Oregon Med. Reporter*,) says also that the statistics of another of our first-class Universities show that five hundred and thirty-five graduates out of nine hundred and ten never marry. Prof. Loomis advances this startling statement: "Men who complete their professional education at or before twenty-one are, as a class, incompetent to perpetuate the race. They have sometimes one, may be two children, but seldom large families. The brain-work of collegiate life so completely deprives the individual of the vitality necessary for the proper development of the generative organs, that our most noted educated men are childless, or have but one or two children. Large portions of our present college students do not even desire married life, and those who do are not able to perpetuate the race." "Important, if true."

Dr. N. M. Logan, of Cincinnati, in a very excellent article in the *Lancet and Observer*, on the Pathology and Treatment of Tuberculosis, recommends to the profession nitric and muriatic acids in the treatment of pulmonary phthisis. Nitric acid he administers in doses of forty drops of the officinal dilution in a couple of ounces of water, immediately after each meal. The muriatic acid he prefers in the combination tr. ferri chloridi, giving this in thirty- or forty-drop doses, sufficiently diluted, half an hour before each meal. He does not expect to supersede by this treatment cod liver oil, suitable food, good hygiene, and other valuable and important measures, nor to remove the tuberculous diathesis, but commends it as having a great superiority over the most recent recognized therapeutics. He considers alcoholic stimulants as being worse than useless, except in cases of extreme exhaustion. A tabulated series of cases is given, sustaining his position. We are ready to indorse this use of the mineral acids, having been accustomed to employ them for this purpose, and inculcate their administration in our teachings at Rush and other medical colleges for many years. These acids furnish the best known succedanea for alchoholic stimulants in about all cases, with the inexpressible advantage that they do not involve the atrocious physical and moral results of habitual alcoholism. Alcohol *per se* has no relation to the cure of tuberculosis or typhoid fever, unless it invigorates nutrition and favors elimination. Neither it nor the mineral acids is a *specific* for these, or even snake bites. Nitric acid, in our experience, is preferable to any of the other mineral acids, both as being more agreeable to the stomach and more energetic in its subsequent influences. But if it does not energize the processes of digestion, repair and elimination in any given case, it will prove useless. The great remedial agencies in tuberculosis are no pitiful drops or magic mixtures, but an entire re-adjustment of all the influences which surround the individual. Mere medication in such cases is temporizing at the best. The patient of phthisical tendency must be warned in time, and, if necessary, put the width of the continent, or the earth's diameter, between him and the concurrent causes which are threatening his existence.

The State Med. Soc. of Ohio passed a resolution petitioning the Legislature to make it a penal offense for any persons but regularly educated physicians to perform the operation of vaccination.——Extreme mercurial treatment of constitutional syphilis is recommended by Prof. LEBERT; he directs suppositories of mercurial ointment, with butter of cocoa.——M. Mehu, Pharmacien of the Necker Hospital, commends this formula for a preservative fluid for specimens: Arsenious Acid, 20 parts; Cryst. Carbolic Acid, 10 parts; Alcohol, 300 parts; Distilied Water, 700 parts. ——A correspondent of the *Chicago Pharmacist* gives a "Quick way of extinguishing mercury." [Why did he not communicate it to an "Eclectic" paper?] He shakes a fluid dram of Tinct. Tolu, with three ounces of Mercury, in a strong two-ounce vial, whereby the fearful metal is readily reduced to a state of minute division, ready for admixture with fatty or other matters.——A correspondent of the *Philadelphia Reporter* has cured a case of "consumption" by excising an elongated uvula.——MR. HENRY LEE treats *nævi* by passing needles under the skin, and producing pressure by twisting above them India rubber threads. The pressure produces sloughing, and then the flaps are brought together, and union favored.——According to the J. G. S. B. the Massachusetts Medical Society proposes to expel from its membership habitual abortionists.——Dr. Luther Parks has retired from the editorial chair of the B. M. & S. Journal which he has ably filled, and is succeeded by Francis H. Brown, M.D., editor, and H. H. A. Beach, associate editor. "Let us have peace."——Catheterism of the larnynx is recommended by Dr. Weinlechner, of Vienna, as a safe and rational resort in some cases which would otherwise require tracheotomy.——A nephew of the recent Sir James Simpson has been appointed to his vacant chair. Much feeling exists at the appointment, as many thought Dr. Duncan should have had the place.——Von Graeffe, the distinguished oculist, is dead.——*The* Newton, whose miraculous cures in this country a few years since will be recollected as akin to the wondrous effects of homœopathic pellets and dilutions, had a brief season of prosperity in England, but this was followed by a sudden decadence of popularity, a mob and threatened lynching; he escaped only by acrobatic feats, which show that his accomplishments are not only spiritual but carnal.——Iodoform is gradually gaining upon the confidence of the profession as a local application to bad conditioned sores, etc. Its internal use is not largely chronicled. Won't somebody report cases enough of its use to start it on its travels?——In the use of Tannin as an antidode for Strychnia, from twenty to twenty-five parts of the former should be used for one of the latter.——Oil of Yellow Sandal Wood is the latest "sure cure" for blennorrhagia.——The *Revue Therap.* gives several cases of relief of abdominal tympanites by puncture with a thin trochar. A neighbor of ours relieved a *cow* of similar trouble by stabbing her with a jack knife.——A correspondent of the *Philadelphia Reporter* records a case of fracture of the tibia from muscular contraction alone. The party was not colored.——Poisoning with Veratrum is recommended in case of poisoning by Opium. In a case (from the *Reporter*) eighteen drops of Norwood's Tincture were given, in two ounces of brandy, to a person deeply narcotized by Opium, and "in an hour by the watch every symptom of Opium poisoning had disappeared.——An English physician recently wrote the following prescription: ℞. Argenti Oxidi gr. xlviij, Morph. Muriat. gr. j, Ext. Gentianæ q. s. M. Make 24 pills and silver them. The lady for whom they were prescribed put the box in her bosom for safe keeping.

The heat of the part caused the compound to explode in about three-quarters of an hour, with resulting destruction of linens and laces, together with a severe burn of the breast. Our contemporaries are exercised at the idea of a similar gastric explosion.——One part of Tannin in sixteen parts of Glycerine is to have dipped in it a bit of lint, and the lint is then to be introduced to the rectum in case of fissure of the anus. Van Holsbek says that he has thus cured fissures which resisted division of the sphincter. ——A new paper has been started in New York as the organ of superfine Positivism, which accepts as waymarks on the high road of progress the crudities and twaddle of Darwin, Spence, *et id omne genus*, believes in "humanity" as the true God, and "regards the worship of an unknowable God as a rank absurdity. Suggests marriages by contract, extending to two or five years only; thinks the old feudal custom of giving the lord of the manor the first privilege of every marriage among his retainers, was useful in improving the blood of the commoners; hints at the propriety of men who are incompetent to beget first class progeny, inviting men of superior strain of blood to the marriage bed; more than intimates that Pierrepont Edwards by his numberless adulteries and fornications did a good thing for Connecticut by distributing a good deal of the blood of his noble father, Jonathan Edwards; believes the 'Social Evil' by no means an unmixed evil, but likely to be ameliorated by polygamy or marriages of limitation," etc., etc., etc. All of which remind us of the old remark, that it is impossible to say anything so absurd but what has been advocated by some of the philosophers. And, again, the follies which astonish us in the medical world—infinitesimals, animal magnetism, womb burning, Newtonism, and hobby-riding in general—find their parallel in other fields of thought and study. When a man once permits himself to swing clear from common sense, there is no measuring the tangent or paradoxical curve he will describe. All healthful progress is achieved not by jumps or saltatory efforts of any kind, even in medical conventions, but by the slower and surer process of growth.

